# Clinical characteristics, treatment, and outcomes of Oxaliplatin-induced immune thrombocytopenia

**DOI:** 10.3389/fimmu.2026.1809508

**Published:** 2026-05-14

**Authors:** Nan Huang, Zheng Liu, Ronghui Li, Haibo Lei, Xiang Liu

**Affiliations:** Clinical Pharmacy, The Central Hospital of Xiangtan, The Affiliated Hospital of Hunan University, Xiangtan, China

**Keywords:** drug-induced thrombocytopenia, immune thrombocytopenia, oxaliplatin, pharmacovigilance, platinum-based chemotherapeutic agents

## Abstract

**Background:**

Although myelosuppression is its common dose-limiting toxicity, oxaliplatin-induced immune thrombocytopenia (OITP), a rare and potentially fatal type II hypersensitivity reaction, is often misdiagnosed as ordinary myelosuppression due to insufficient clinical awareness.

**Methods:**

Relevant literature published up to December 31, 2025 were retrieved from databases including PubMed, Scopus, Embase, Web of Science, Cochrane Library, Wanfang Medical Database and China National Knowledge Infrastructure (CNKI). Cases confirmed as OITP were strictly screened for retrospective analysis.

**Results:**

Among the 40 included patients, the median age was 59 years (range, 36–83 years), with a significant female predominance (65.0%). The main primary disease was colorectal cancer (92.5%), and the most commonly used chemotherapy regimen was FOLFOX (77.5%). OITP onset was characterized by a distinct latent period, with a median time to onset of the 9th chemotherapy cycle (range, 2–28 cycles). The clinical manifestations were severe: 82.5% of patients had a platelet count drop to < 25×10^9^/L, with a median nadir of only 6×10^9^/L. Bleeding symptoms were evident in 65.0% of patients, and 52.5% of patients had accompanying systemic hypersensitivity symptoms such as fever and chills. Immunological tests showed that oxaliplatin-dependent platelet antibodies were detected in 48.4% of patients. In terms of treatment, oxaliplatin was discontinued in all patients (100%), combined with platelet transfusion (62.5%), glucocorticoids (55.0%), intravenous immunoglobulin (20.0%) and plasma exchange (12.5%). The overall recovery rate was 92.5%, with a median recovery time of 7 days, and the mortality rate reached 7.5%.

**Conclusions:**

OITP is a severe immune complication occurring after cumulative exposure to oxaliplatin, often accompanied by systemic allergic symptoms. Clinicians should be alert to sudden severe thrombocytopenia after long-term oxaliplatin administration, and permanent discontinuation of oxaliplatin is strongly advised once OITP is diagnosed. In clinical practice, glucocorticoids and platelet transfusion are the main empirical treatment options for OITP, and plasma exchange may be a potential empirical salvage intervention for patients with refractory or life-threatening OITP, while the efficacy of these treatments has not been validated by comparative data.

## Introduction

Since its approval in the 1990s, oxaliplatin, a third-generation diaminocyclohexane (DACH) platinum compound, has revolutionized the treatment landscape of gastrointestinal malignancies (1). Its unique pharmacological mechanism lies in inducing tumor cell apoptosis by forming intra- and interstrand DNA cross-links, which block DNA replication and transcription (2). At present, oxaliplatin-based combination chemotherapy regimens, such as FOLFOX (oxaliplatin + fluorouracil + leucovorin) and XELOX (oxaliplatin + capecitabine), have become the standard regimens for adjuvant chemotherapy of stage II high-risk and stage III colorectal cancer, as well as first-line treatment for advanced metastatic colorectal cancer (3, 4).

With the widespread clinical application of oxaliplatin and the prolonged survival of patients, the problem of its cumulative toxicity has become increasingly prominent. In addition to the well-known dose-limiting peripheral neurotoxicity (with an incidence of approximately 15%-20% for grade 3/4 neurotoxicity), hematological toxicity also cannot be ignored (5–7). Thrombocytopenia is a common complication in oxaliplatin-treated patients, and its pathogenesis is mainly attributed to myelosuppression. This type of thrombocytopenia caused by the direct cytotoxic effect of chemotherapeutic agents on megakaryocyte precursors is usually dose-dependent, develops slowly, and mostly recovers spontaneously during the chemotherapy interval (8). Unlike common myelosuppression, oxaliplatin-induced immune thrombocytopenia (OITP) is a special type of thrombocytopenia with acute onset, complex mechanism and extremely high risk (9). It belongs to the category of drug-induced immune thrombocytopenia (DITP), which is usually mediated by drug-dependent antibodies. Such antibodies (mostly IgG type) bind with high affinity to platelet membrane glycoproteins (such as GPIIb/IIIa or GPIb/IX complexes) only in the presence of oxaliplatin, leading to rapid destruction of sensitized platelets in the mononuclear phagocyte system (10, 11).

Studies have shown that the incidence of oxaliplatin-related severe thrombocytopenia is 3-4%, and it increases significantly with the increase of cumulative courses (12). Despite the high mortality rate of OITP, clinical awareness of this condition remains inadequate (13). Due to its insidious and acute onset, and its frequent occurrence in patients who have tolerated multiple cycles of chemotherapy, it is easily misdiagnosed as consumptive thrombocytopenia caused by tumor progression or severe myelosuppression. In addition, there is no unified guideline for the treatment of OITP. Although the American Society of Hematology (ASH) has issued detailed treatment guidelines for primary immune thrombocytopenia (ITP), the intervention strategies for chemotherapy-induced secondary ITP, especially for oxaliplatin, rely mostly on case reports and empirical treatment (14). This study investigated the clinical characteristics, treatment regimens, diagnostic methods and prognostic outcomes associated with oxaliplatin-induced ITP, aiming to promote the prudent clinical application of oxaliplatin.

## Methods

### Literature search strategy

Data sources included PubMed, Scopus, Embase, Web of Science, Cochrane Library, Wanfang Medical Database and China National Knowledge Infrastructure (CNKI). The retrieval time is from the inception of each database up to December 31, 2025. Search keywords were a combination of Medical Subject Headings (MeSH terms) and free text words. The specific search string was constructed as follows: (“Oxaliplatin” OR “OXA” OR “Platinum compounds”) AND (“Thrombocytopenia” OR “Immune Thrombocytopenia” OR “ITP” OR “Purpura, Thrombocytopenic, Idiopathic” OR “Purpura, Thrombocytopenic”).

### Inclusion and exclusion criteria

#### Inclusion criteria

(1) Patients diagnosed with malignant tumors and receiving oxaliplatin-containing chemotherapy regimens. (2) Patients with sudden and severe thrombocytopenia (platelet count < 100 × 10^9^/L) with a clear temporal correlation with oxaliplatin administration. (3) Literature providing complete core clinical data including age, gender, diagnosis, number of chemotherapy cycles, the lowest platelet count, therapeutic measures and outcomes.

#### Exclusion criteria

(1) Thrombocytopenia clearly caused by other reasons such as sepsis, disseminated intravascular coagulation, bone marrow metastasis or combined other hematological diseases. (2) Only presenting with chronic and progressive myelosuppression without acute onset characteristics. (3) Literature with severe missing data that cannot extract key variables. (4) Studies that only reported aggregated data without providing detailed individual patient characteristics. (5) Duplicate publications or review articles. (6) Animal experiments or mechanism research articles.

### Data extraction

Based on a pre-designed structured data extraction form, two researchers independently extracted data, including: (1) Demographic characteristics: gender, age, nationality. (2) Tumor and treatment background: primary tumor type, specific chemotherapy regimen (FOLFOX, XELOX, etc.), concomitant medication, past medical history. (3) Onset characteristics: number of chemotherapy cycles at onset (latent period), initial symptoms (bleeding site, systemic reactions). (4) Laboratory indicators: the lowest platelet count, white blood cell count, hemoglobin level, antibody test results (drug-dependent antibodies, direct antiglobulin test [DAT]). (5) Treatment and prognosis: whether to discontinue the drug, specific drug therapy (hormones, intravenous immunoglobulin), mechanical therapy (plasma exchange, blood transfusion), recovery time, survival status and response after rechallenge.

### Diagnostic criteria and antibody detection

The detection of oxaliplatin-dependent platelet antibodies followed standard laboratory protocols established for identifying DITP (10). In this process, the patient’s serum is co-incubated with normal type O platelets in the presence of oxaliplatin. A specific binding reaction is confirmed if the fluorescence intensity produced by the binding of immunoglobulin G antibodies is twice or more that of the normal control sample, provided that no such binding reaction occurs in the absence of oxaliplatin.

### Data processing and statistical analysis

SPSS 22.0 statistical software was used for data summary and analysis. Measurement data conforming to normal distribution were expressed as mean ± standard deviation; measurement data with non-normal distribution were described by median and range (minimum-maximum). Count data were expressed as frequency (n) and percentage (%).

## Results

### Epidemiological characteristics

**Figure 1:**
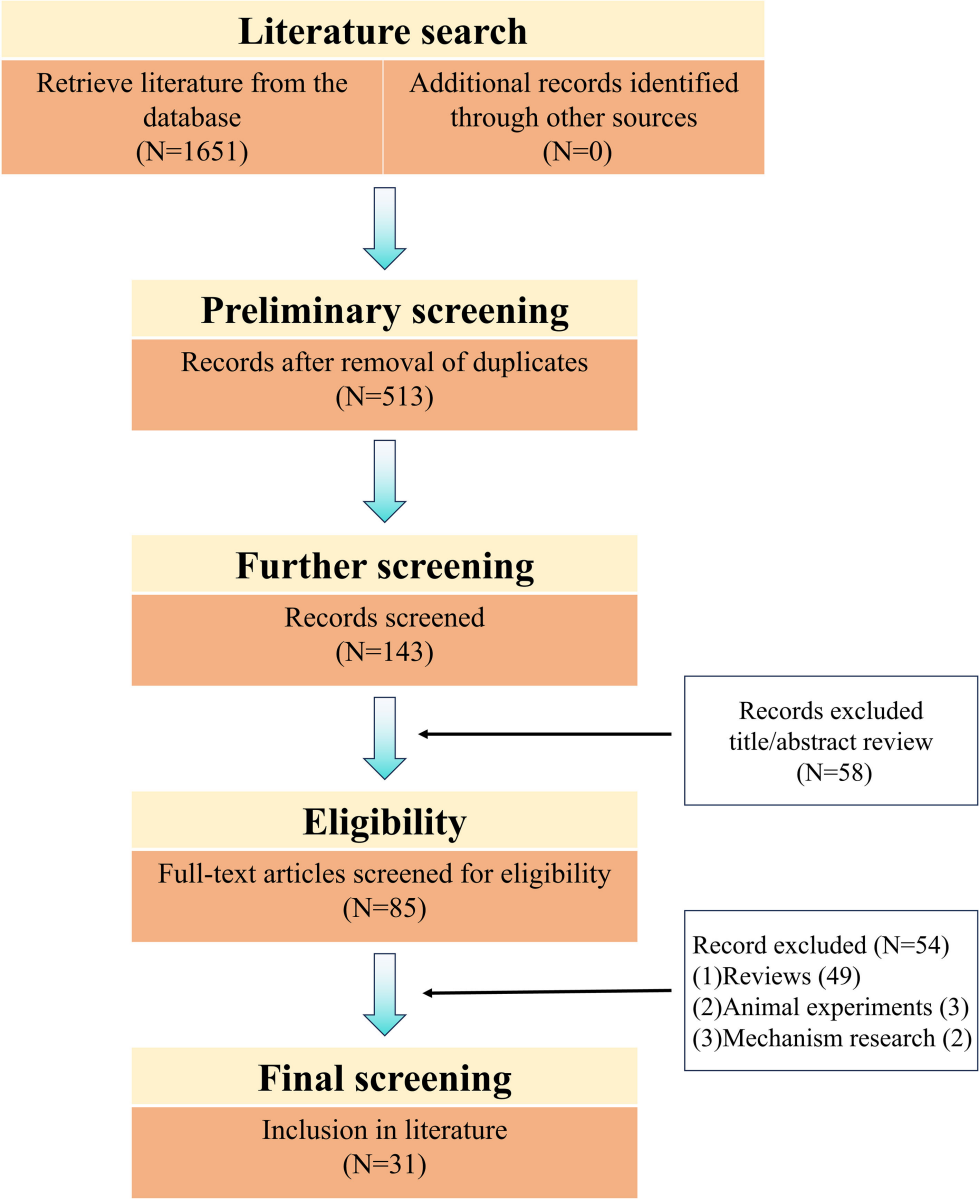
Literature screening flow chart.

In the initial stage of the study, a total of 1651 relevant literature records were obtained through database retrieval combined with manual screening; after excluding duplicate literature and conducting hierarchical screening of titles, abstracts and full texts, 31 studies were finally identified for subsequent analysis (**Figure 1**) (10, 11, 15–43). A total of 40 patients with OITP meeting the criteria were included in this study (**Table 1**). The disease was more common in female patients, with 26 female patients (65.0%) and 14 male patients (35.0%). This gender ratio (female: male ≈ 1.86:1) was opposite to the trend of a slightly higher incidence of colorectal cancer in males. The median age of the patients was 59 years, with a wide age range (36–83 years), which was consistent with the high-incidence age group of colorectal cancer. Cases were mainly concentrated in Europe, America and East Asia, with the most reports from the United States (16 cases, 40.0%), followed by France (5 cases, 12.5%). Among Asian countries, there were 2 reported cases each from South Korea, Taiwan, China and Turkey. This reflects the global prevalence of this complication, and may also be related to the frequency of oxaliplatin use and the improvement of pharmacovigilance systems in different regions. The vast majority of patients (92.5%) were treated for colorectal cancer, followed by gastric cancer (5.0%) and pancreatic cancer (2.5%). In terms of specific chemotherapy regimens, the FOLFOX regimen was the most common (77.5%), and the XELOX regimen accounted for 15.0%. Notably, most patients (85.0%) were treated with a combination of 5-FU and/or leucovorin, and some were combined with bevacizumab or cetuximab, which increased the complexity of differential diagnosis. However, in studies with immunological confirmation, oxaliplatin was confirmed as the only sensitogen. The latent period (sensitization period) of onset is a key clinical feature of OITP, which does not occur at the first administration. Data showed that the median time to onset was the 9th cycle (range, 2–28 cycles). This long latent period strongly supports the immune sensitization mechanism—the body requires multiple exposures to the antigen (oxaliplatin and its metabolites) to produce a sufficient titer of high-affinity antibodies. A portion of patients (27.5%) had underlying diseases such as hypertension, diabetes, heart failure or anemia, which may have impaired the patients’ compensatory capacity for acute bleeding events. The shortest time from the start of oxaliplatin infusion to the occurrence of OITP was 15 minutes, the longest was 48 hours, with a median of 10 hours.

**Table 1:** Basic characteristics of 40 patients with OITP.

Parameter	Classification	Value
Sex	Female	26 (65.0%)
	Male	14 (35.0%)
Age	Years	59 (36, 83) ^b^
Country	USA	16 (40.0%)
	France	5 (12.5%)
	Israel	3 (7.5%)
	Italy	3 (7.5%)
	Taiwan, China	2 (5.0%)
	Czech Republic	2 (5.0%)
	Korea	2 (5.0%)
	Turkey	2 (5.0%)
	Norway	2 (5.0%)
	Brazil	1 (2.5%)
	UK	1 (2.5%)
	Switzerland	1 (2.5%)
Regimen	FOLFOX	31 (77.5%)
	XELOX	6 (15.0%)
	Other Oxaliplatin-based	3 (7.5%)
Indication	Colorectal cancer	37 (92.5%)
	Gastric cancer	2 (5.0%)
	Pancreatic cancer	1 (2.5%)
Latency Time to Onset	Cycles (25) ^a^	9 (2, 28) ^b^
	Hours (25) ^a^	10 (0.25, 48) ^b^
Medical history	Hypertension, Diabetes, Heart failure, Neuropathy, Anemia	11 (27.5%)
Concomitant medications	5-FU/Leucovorin	34 (85.0%)
	Capecitabine	6 (15.0%)
	Bevacizumab	11 (27.5%)
	Cetuximab	2 (5.0%)

^a^Represents the number of patients in whom information was provided; ^b^Median (minimum, maximum).

### Clinical symptoms and manifestations

Unlike the fatigue and progressive bleeding caused by chronic myelosuppression, OITP is characterized by fulminant clinical manifestations. **Table 2** summarizes the symptoms and laboratory test results of the 40 patients. More than half of the patients (65.0%) had obvious bleeding events, including gastrointestinal bleeding (melena, hematemesis), gross hematuria, epistaxis and gingival bleeding. Such bleeding often occurs during or within a few hours after infusion, which is of extremely high warning significance. Skin purpura or petechiae, a typical sign of severe thrombocytopenia, was observed in 25.0% of patients. Systemic symptoms such as fever, chills, back pain and fatigue were present in 52.5% of patients. These symptoms are key clues for differentiating ITP from myelosuppression.

**Table 2:** Clinical symptoms and laboratory test characteristics of 40 patients.

Parameter	Classification	Value
Signs and symptoms	Purpura/Petechia	10 (25.0%)
	Bleeding: GI bleeding, Hematuria, Epistaxis, Gingival bleeding, etc.	26 (65.0%)
	Systemic: Fever, Chills, Back pain, Fatigue	21 (52.5%)
Laboratory tests		
Platelet	Median (10^9^/L)	6 (0, 80) ^b^
	< 25×10^9^/L	33 (82.5%)
	≥ 25×10^9^/L	7 (17.5%)
WBC (28) ^a^	Normal range	16 (57.1%)
	Decrease	9 (32.1%)
	Increase	3 (10.7%)
Hemoglobin (32) ^a^	Normal range	8 (25.0%)
	Decrease	24 (75.0%)
Antibodies (31) ^a^	Oxaliplatin-dependent platelet antibodies (+)	15 (48.4%)
	DAT (+)	16 (51.6%)
	PLT (+)	1 (3.2%)

^a^Represents the number of patients in whom information was provided; ^b^Median (minimum, maximum); DAT (+): an independent finding of red blood cell sensitization.

### Laboratory test characteristics

**Figure 2:**
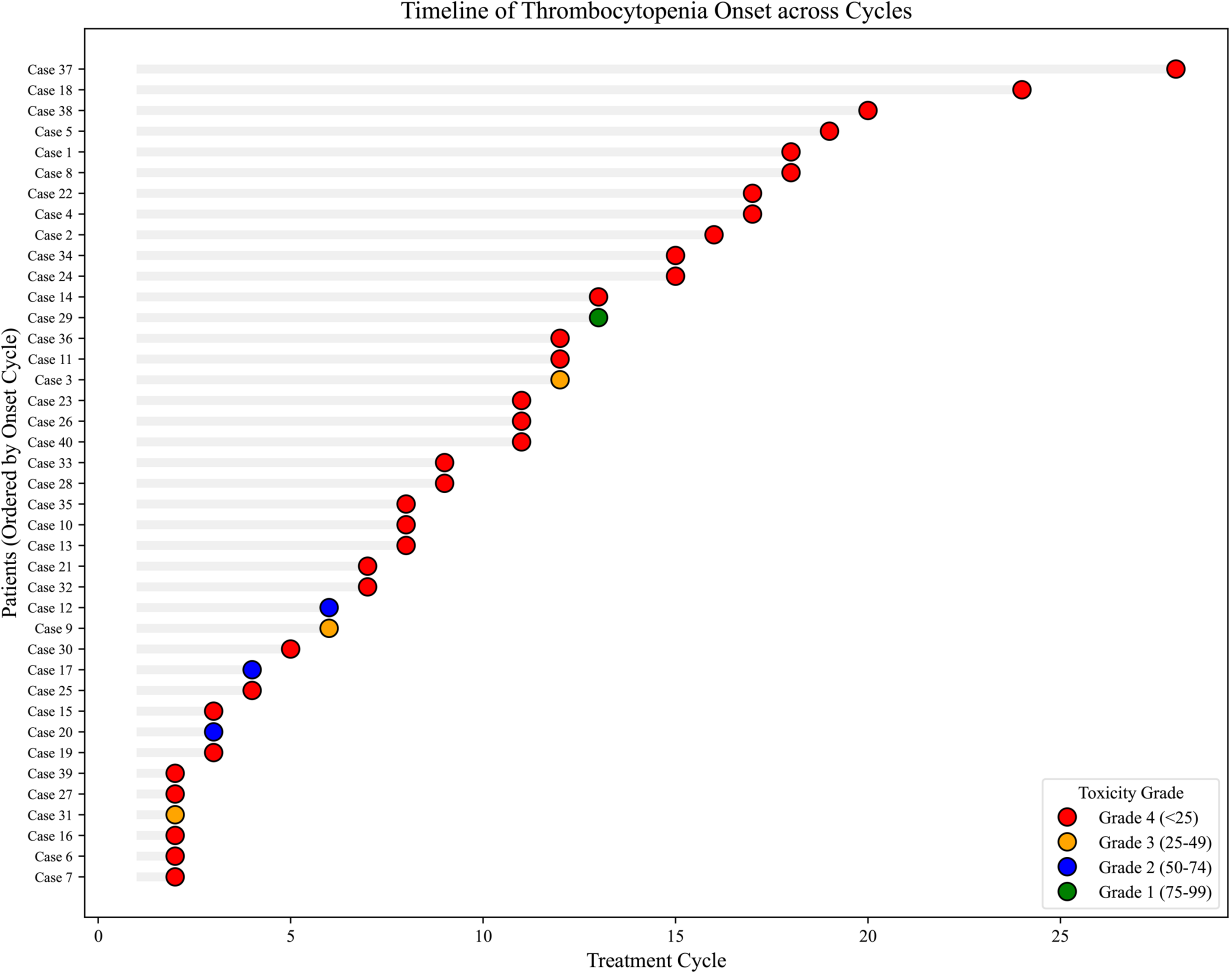
Swimmer plot illustrating the timeline of OITP onset across treatment cycles. This swimmer plot displays the temporal distribution of OITP onset for individual cases (n=40). Each horizontal gray bar represents a unique patient, with the x-axis indicating the number of oxaliplatin treatment cycles. The colored circles at the end of each bar mark the specific cycle of OITP onset. The severity is graded according to the NCI-CTCAE v5.0 criteria: red circles denote Grade 4, orange for Grade 3, blue for Grade 2, and green for Grade 1. The plot highlights a broad onset window (ranging from cycle 2 to 28) and a high prevalence of severe Grade 4 events, underscoring the need for continuous hematological monitoring throughout the entire course of oxaliplatin therapy.

Laboratory data revealed an astonishing rate of platelet destruction. The median platelet count at onset was only 6×10^9^/L (range, 0-80×10^9^/L), far lower than the trough value of conventional chemotherapy-induced myelosuppression. As many as 82.5% of patients had a platelet count below 25×10^9^/L (**Figure 2**). Although OITP primarily affects platelets, 75.0% of patients also exhibited a decrease in hemoglobin levels, indicating the presence of anemia, which may be multifactorial in oncologic patients, including bleeding, disease-related factors, or treatment effects. The white blood cell count remained normal in most patients (57.1%), and even a small number of patients (10.7%) had an elevated white blood cell count, which helps to rule out pancytopenia or severe bone marrow failure. Among patients who underwent specific antibody testing, 48.4% were positive for oxaliplatin-dependent platelet antibodies, which is the gold standard for the diagnosis of OITP. In addition, 51.6% of patients had a positive DAT, further confirming the immune-mediated pathological mechanism. Due to the lack of elution testing in the original case reports, the drug-dependency of DAT positivity could not be confirmed.

### Subgroup analysis: comparison of oxaliplatin-dependent platelet antibodies confirmed and unconfirmed cases

To further explore the impact of antibody status on clinical presentation, we compared patients with confirmed oxaliplatin-dependent antibodies (n=15) and those without confirmatory testing (n=25). As summarized in **Table 3**, both subgroups had similar median onset cycles (8 vs. 9) and rates of systemic symptoms (46.7% vs. 48.0%). However, the confirmed group exhibited a lower median platelet nadir (4.0×10^9^/L vs. 8.0×10^9^/L), indicating more severe thrombocytopenia. These data suggest that while antibody confirmation correlates with greater platelet destruction, the overall clinical phenotype remains consistent across groups.

**Table 3:** Comparison of clinical characteristics between oxaliplatin-specific antibody confirmed and unconfirmed OITP cases.

Characteristics	Confirmed n=15	Unconfirmed n=25
Gender (Male/Female)	3/12	11/14
Median age (Range), years	58 (38–79)	60 (36–83)
Median onset cycle (Range)	8 (2–28)	9 (2–24)
Median nadir platelet count (Range), ×10^9^/L	4.0 (0.0–66.0)	8.0 (0.9–80.0)
Systemic symptoms (n, %)	7 (46.7%)	12 (48.0%)

### Treatment and prognosis

The treatment strategy for OITP was mainly to rapidly block the immune response and provide supportive care. **Table 4** shows the specific therapeutic measures and outcomes. Oxaliplatin was immediately discontinued in all patients (100%), which was the primary life-saving measure. In terms of drug therapy, 55.0% of patients received systemic glucocorticoid therapy to inhibit the immune response. Given the critical condition, 62.5% of patients received emergency platelet transfusion, which is a necessary measure to prevent fatal bleeding despite the potentially limited efficacy in the presence of immune destruction. Intravenous immunoglobulin (IVIG) was used in 20.0% of patients, and plasma exchange was performed in 12.5% of critically ill patients to eliminate pathogenic antibodies and immune complexes in the blood. The vast majority of patients (92.5%) achieved a normal platelet count after treatment, with a median recovery time of 7 days (range, 3–60 days). This characteristic of rapid recovery is consistent with the pharmacokinetics of the drug—the antibody-mediated destruction ceases with the clearance of the drug from the body. However, 3 patients (7.5%) unfortunately died, mainly due to uncontrollable intracranial hemorrhage or massive gastrointestinal bleeding, accompanied by multiple organ failure. Data showed that all 5 patients who attempted rechallenge with oxaliplatin (100%) experienced a relapse.

**Table 4:** Treatment measures and clinical outcomes of 40 patients.

Parameter	Classification	Value
Treatment	Withdraw Oxaliplatin	40 (100%)
	Systemic steroids	22 (55.0%)
	Platelet transfusion	25 (62.5%)
	IVIG	8 (20.0%)
	Plasma exchange	5 (12.5%)
Outcome	Recovery	37 (92.5%)
	Death	3 (7.5%)
Recovery time (28) ^a^	days	7 (3, 60) ^b^
Rechallenge (5) ^a^	Relapse	5 (100%)
	No relapse	0 (0%)

^a^Represents the number of patients in whom information was provided; ^b^Median (minimum, maximum).

## Discussion

Chemotherapy can cause a variety of adverse events, but immune-mediated thrombocytopenia occurs relatively infrequently (44). A comprehensive review of platinum-based chemotherapy shows that the incidence of oxaliplatin-induced hematological adverse events is approximately 10%, among which severe immune-mediated cases account for only 3-4% (12). Most of these events usually manifest after multiple cycles of chemotherapy. Specifically, the median time to onset of oxaliplatin-induced ITP is the 9th cycle, which is consistent with other immune-mediated adverse events. Oxaliplatin-induced thrombocytopenia can also be complicated with acute hemolysis and acute immune pancytopenia (11, 29). In our research, most patients presented with bleeding symptoms, while a subset also exhibited systemic manifestations such as fatigue and fever. Additionally, symptoms such as back pain were observed in some patients; however, the underlying mechanisms remain unclear. Given the lack of specific laboratory evidence, hemolysis could not be confirmed in our study, and anemia in these patients is more likely to be multifactorial, including bleeding and disease- or treatment-related factors.

The exact pathophysiological mechanism of OITP remains to be fully elucidated. Current evidence suggests that oxaliplatin-dependent platelet antibodies bind to platelet membrane glycoproteins, most commonly the GPIIb/IIIa complex, although GPIb/IX and GPIa/IIa have also been described as potential antigenic targets (10, 11, 41). Once platelets are opsonized, their clearance is thought to occur predominantly through Fcγ receptor (FcγR)-mediated phagocytosis by macrophages in the reticuloendothelial system (45). In addition to this pathway, complement activation may further contribute to platelet destruction through C3b deposition and, in some cases, membrane attack complex (MAC) formation (46). Such immune activation, including enhanced macrophage phagocytic activity and complement cascade engagement, may be associated with cytokine release (47). This process could partially contribute to systemic manifestations such as fever, chills, and pain observed in some patients. However, these mechanisms have not been directly demonstrated in most reported OITP cases, including our cohort, and should therefore be considered biologically plausible rather than definitively established. Further mechanistic studies are required to clarify the relative contributions of FcγR-mediated clearance, complement activation, and other immune pathways in OITP. Clinical observations suggest that re-exposure to oxaliplatin in sensitized individuals may activate immune memory, potentially leading to a rapid and enhanced immune response (18).

The clinical diagnosis of drug-induced thrombocytopenia should be carried out in accordance with established standards (48). Unlike other drugs that require continuous administration, oxaliplatin is administered intermittently in chemotherapy regimens. This administration characteristic makes it easier to clarify the causal relationship between oxaliplatin and thrombocytopenia, and drug rechallenge test is unnecessary and not recommended in clinical practice. Laboratory confirmation can be achieved by flow cytometry, which is centered on detecting the presence of oxaliplatin-dependent antibodies in the patient’s serum. Although this detection method is not widely available at the time of patient presentation, it can provide an important reference for the diagnosis of oxaliplatin-induced acute thrombocytopenia and clinical drug withdrawal decisions. If this test cannot be performed, diagnosis can also be made based on comprehensive clinical judgment. It should be noted that oxaliplatin is usually administered in combination with other chemotherapeutic agents, which may confound the immune response. In our study, most cases involved the combined use of oxaliplatin with 5-FU and leucovorin. According to literature reports, the above reaction antibodies can only react with platelets in the presence of oxaliplatin, but not in the presence of 5-FU or leucovorin. Furthermore, our literature review yielded no evidence of ITP induced by 5-FU or leucovorin alone, reinforcing oxaliplatin as the primary etiological agent. In addition, nearly half of the cases collected in our study were positive for oxaliplatin antibodies, which also verified that oxaliplatin induced ITP.

The core of the treatment for DITP is the immediate discontinuation of the suspected causative drug. Clinically, priority should be given to discontinuing new drugs initiated within 5–10 days before the onset of the disease; for patients receiving multiple drugs in combination, newly added drugs in the recent period can be discontinued first, or drugs can be discontinued sequentially according to the prior probability of drug-induced diseases. If necessary, drugs with different chemical structures but equivalent pharmacological effects can be used instead to ensure the treatment of the underlying disease (49). Given that the detection of drug-dependent antibodies (DDAbs) is time-consuming and not yet popularized, the decision of drug withdrawal should be based on comprehensive clinical judgment without waiting for test results (50).

In symptomatic supportive care, platelet transfusion is only recommended for severe thrombocytopenia patients with “wet purpura” (such patients have a high risk of intracranial hemorrhage), but it is ineffective when the plasma still contains the causative drug or its metabolites, and the efficacy of this regimen lacks confirmation from formal clinical studies. Glucocorticoids are routinely used in the management of ITP; however, their specific efficacy in treating OITP requires further validation (51). For patients with severe bleeding, high bleeding risk or rare cases with persistent symptoms for several weeks, advanced treatment can be adopted: the conventional dose of IVIG is 1g/kg body weight. Serum samples should be collected for DDAbs detection before medication (IVIG can interfere with immunoassays, and sampling should be performed at least 48 hours after medication), which can accelerate platelet recovery but the benefit is only potential. Plasma exchange is only used for the above rare refractory cases, and its efficacy is also unclear (49, 50). Notably, although the efficacy of glucocorticoid and/or IVIG therapy has not been clinically confirmed, guidelines from the American Society of Clinical Oncology (ASCO) clearly recommend the above drugs for the treatment of ITP (52).

In most DITP patients, the drug and its metabolites can be cleared within a few days after discontinuation of the causative drug, and the platelet count usually rises 1–2 days after admission. Although DDAbs detection has no practical value for the diagnosis and treatment of the acute phase, it can identify the causative drug to guide subsequent avoidance. In clinical practice, empirical therapy should be minimized; instead, management strategies should be individualized based on the patient’s bleeding risk and the expected duration of symptoms. Re-exposure to the suspected causative drug is contraindicated. Clinical observations suggest that re-exposure to oxaliplatin in sensitized individuals may activate immune memory, potentially leading to a rapid and enhanced immune response (18). This risk is strongly corroborated by our findings, where all 5 patients who underwent oxaliplatin rechallenge experienced a recurrence of severe thrombocytopenia (100% relapse rate). Therefore, to ensure patient safety, permanent discontinuation of oxaliplatin is strongly advised once OITP is diagnosed, and drug rechallenge should be avoided.

A recent prospective cohort study by Vázquez-Revuelta et al. complements our case-based analysis of OITP (53). In their cohort, acute immune thrombocytopenia was the most common type II hypersensitivity reaction (56%), and all patients developed thrombocytopenia—consistent with our findings. They reported a mean cumulative oxaliplatin cycle of 20, but in the retreatment population (88% of their cohort), the index reaction occurred at a median of cycle 9 of the current treatment, which aligns closely with our median onset of cycle 9 (range 2–28). Drug-dependent antibodies were detected in 75% of their tested patients. Notably, they successfully re-exposed five selected patients under strict protocols with manageable breakthrough reactions, whereas all five rechallenged patients in our study relapsed (100%), highlighting that re-exposure may be feasible only in highly specialized centers with rigorous monitoring. Additionally, elevated interleukin-6 without tryptase elevation emerged as a potential biomarker to differentiate type II from type I hypersensitivity. Collectively, this cohort study enriches our understanding of OITP by providing prospective data on diagnosis, antibody testing, and carefully managed re-exposure.

## Limitations of the study

The present study has the following limitations: 1) Severe publication bias, as published case reports tend to include severe, fatal, or atypical cases, while mild or asymptomatic cases are underreported, which may lead to an overestimation of the severity and mortality of oxaliplatin-induced immune thrombocytopenia (OITP) in this study; 2) Heterogeneity in the quality of included case reports, with variations in the completeness of clinical data and standardization of diagnostic methods across different reports, which may affect the comprehensiveness and accuracy of the aggregated descriptive data; 3) Lack of standardized antibody testing methodologies, as the detection methods and criteria for drug-dependent antiplatelet antibodies and DAT vary across reports, potentially affecting the statistical results of antibody positivity rates; 4) Confounding effects of combination chemotherapy, as most patients received oxaliplatin in combination with 5-FU, bevacizumab, or other agents, and the potential synergistic effects of combination chemotherapy on OITP cannot be completely excluded, adding complexity to the descriptive analysis of clinical characteristics; 5) Potential misclassification bias, due to the overlapping clinical manifestations among OITP, oxaliplatin-induced immune syndrome (OIIS), and drug-induced thrombotic thrombocytopenic purpura (TTP), making it impossible to completely rule out partial misclassification; 6) Absence of structured bleeding severity grading, as the original case reports did not adopt standardized bleeding severity scales, preventing a quantitative analysis of the relationship between bleeding severity and treatment/prognosis; 7) Uncertainty in mortality attribution, as the 7.5% mortality rate in this study was associated with intracranial hemorrhage/gastrointestinal bleeding, but due to incomplete published data, the potential contributions of underlying malignancy, combination chemotherapy, and comorbidities to patient death cannot be accurately distinguished. Nevertheless, this study summarizes the available evidence and may help clinicians to early identify and appropriately manage oxaliplatin-induced immune-related thrombocytopenia.

## Conclusions

OITP is a rare but severe chemotherapy-related adverse reaction. The disease is characterized by a long latent period of onset, a high incidence of bleeding symptoms and an extremely low platelet count. Timely discontinuation of oxaliplatin combined with systemic glucocorticoid and platelet transfusion is the main treatment strategy, and most patients can achieve clinical recovery. Clinicians should be alert to the occurrence of ITP during oxaliplatin chemotherapy, and conduct timely diagnosis and intervention to improve patient prognosis. Future research should focus on identifying predictive biomarkers (such as specific HLA genotypes) to identify high-risk populations before chemotherapy, thereby achieving truly individualized and precise treatment.

## Data Availability

The original contributions presented in the study are included in the article/supplementary material. Further inquiries can be directed to the corresponding authors.
